# Alexithymia, anxiety and depression in patients with psoriasis: a case–control study

**DOI:** 10.1186/s12991-014-0038-7

**Published:** 2014-12-10

**Authors:** Panagiota Korkoliakou, Christos Christodoulou, Anargyros Kouris, Evgenia Porichi, Vasiliki Efstathiou, Eythymia Kaloudi, Anna Kokkevi, Nikolaos Stavrianeas, Charalabos Papageorgiou, Athanasios Douzenis

**Affiliations:** Second Department of Psychiatry, University of Athens Medical School, “Attikon” University General Hospital, Athens, 12462 Greece; Second Department of Dermatology, University of Athens Medical School, “Attikon” University General Hospital, Athens, 12462 Greece; University Mental Health Research Institute (UMHRI), Athens, 15601 Greece

**Keywords:** Alexithymia, Anxiety, Depression, Psoriasis, TAS-20

## Abstract

**Background:**

Alexithymia, the difficulty in describing or recognizing emotions, has been associated with various psychosomatic pathologies including psoriasis. The aim of this study was to examine the prevalence of alexithymia and its association with anxiety and depression in patients with psoriasis compared with healthy participants, while taking into consideration demographic and clinical variables.

**Methods:**

One hundred and eight psoriatic patients and 100 healthy participants from the general population completed the Toronto Alexithymia Scale (TAS-20) and the Hospital Anxiety and Depression Scale (HADS). The severity of patients’ psoriasis was clinically assessed using the Psoriasis Area and Severity Index (PASI).

**Results:**

Psoriatic patients had higher levels of alexithymia compared with healthy participants. While a rather high rate of psoriatic patients presented anxiety and depression as defined by the HADS, the differences that were found in comparison with the control group were not significant. Neither alexithymia nor its dimensions, difficulty in identifying feelings (DIF), difficulty in describing feelings (DDF) and externally oriented thinking (EOT), were associated with gender or psoriasis severity. Age was associated only with EOT, which was independent of depression and anxiety. Higher anxiety and depression were connected with higher alexithymia and DIF, while higher anxiety with higher DDF as well.

**Conclusions:**

The alexithymia prevalence was higher in psoriatic patients than that in healthy participants, while it was positively correlated with anxiety and depression. Difficulty in identifying feelings was connected with both anxiety and depression, whereas difficulty in describing them was only with anxiety. Finally, externally oriented thinking was predicted only from age.

## Background

The term alexithymia was coined by Sifneos [1] in order to describe the deficiency in understanding, processing and describing emotions [1]. Alexithymia is considered a personality trait that consists of reduced symbolic thought, restricted fantasy life, externally oriented cognitive thinking, difficulty in distinguishing feelings from bodily sensations and inadequacy in intuition and empathy [2]. The authors of relevant studies have differentiated between primary alexithymia, which is developed prematurely in the first period of life due to fixations and structural neurological deficits and secondary alexithymia which arises after trauma or severe pathology.

In the literature, alexithymia is considered to act as a triggering factor of general susceptibility to diseases, among other variables [2]. A view of alexithymia is that it constitutes a risk factor for psychiatric and behavioural problems and also for the development, maintenance or exacerbation of medical or physical health problems [3].

The majority of studies regarding the relationship between alexithymia, depression and anxiety conclude that they are strongly related [4,5]. Alexithymia is not unusual among patients with certain skin disorders [6]. Its presence has been incriminated in the genesis and in the maintenance of psoriasis, which has been classified among psychosomatic illnesses [7]. Psoriasis is a chronic, inflammatory, relapsing skin disease. Both genetic and environmental factors are believed to play an important role in the pathogenesis of the disorder [8]. Psoriasis affects 2%–3% of the population and has an equal distribution between the genders, and its onset may occur at any age. Incidents of psoriasis have been connected to pathological states of stress and anxiety such as impatience, depression and pathological worry [9]. Despite the existence of the psychosomatic hypothesis, only a few researchers have studied the association between alexithymia and psoriasis and most of them without using a control group or in case they did; this consisted of a relatively small number of participants [9,10]. In spite of the aforementioned limitation, the majority of studies have found a significant difference in the levels of alexithymia between patients with psoriasis and healthy individuals [11-13]. Furthermore, most studies concerning alexithymia in psoriatic patients have not taken into account important factors, which are associated with both psoriasis and alexithymia, such as depression or anxiety, or they have evaluated them using psychometric tools that contain somatic aspects which may also be common in psoriasis [11]. In Greece, studies have mainly focused on the relationship between psoriasis and depression [14].

Considering the paucity of relevant studies in the scientific literature as well as the aforementioned limitations of the earlier studies, the purposes of the present study are (a) to examine the prevalence of alexithymia among patients with psoriasis, in comparison with healthy individuals from the general population, and (b) to assess the relationship between depressive and anxiety symptoms and alexithymia, as well as its three dimensions, difficulty in indentifying feelings (DIF), difficulty in describing feelings (DDF) and externally oriented thinking (EOT), by taking into account demographic and clinical data of the patients.

## Methods

### Sample and procedure

The list of scheduled appointments of the dermatologic clinic was used in order to recruit 108 patients with confirmed diagnosis of chronic plaque psoriasis, with a minimum of 6 years of education and ability to comprehend the Greek language. Patients were asked to participate in the study between January 2009 and October 2010 in “Attikon” University General Hospital. The Toronto Alexithymia Scale (TAS-20) and the Hospital Anxiety and Depression Scale (HADS) were administered to the psoriatic patients, while demographic and clinical data were collected as well. Main exclusion criteria were co-morbidity with a diagnosed physical or psychiatric disorder and receiving medication that could have affected their mental condition, including illegal substances and alcohol.

As a control group, we used 100 healthy individuals from the general population, matched for age and gender with the patients, who were recruited during the same time period. They were included provided that they were not diagnosed with any physical or/and psychiatric disorders, had a minimum of 6 years of education and ability to comprehend the Greek language. The TAS-20 and the HADS were also completed by the control group, and demographic data were obtained as well.

The participation of both patients and healthy controls was voluntary, without any financial compensation. The research protocol was approved by the ethics committee of the “Attikon” University General Hospital. All participants provided written informed consent, and the study was carried out in accordance with the declaration of Helsinki.

### Assessment instruments

Alexithymia was assessed using the 20-item self-report TAS-20. The scale includes 20 items rated on a 5-point Likert scale, ranging from 1 (strongly disagree) to 5 (strongly agree). The total score of the scale ranges from 0 to 100. It consists of three subscales, measuring the difficulty in identifying feelings (DIF), the difficulty in describing feelings (DDF) and the externally oriented thinking (EOT), respectively. According to the accepted standard, a total score above 61 indicates alexithymia, a score between 52 and 60 suggests intermediate/borderline alexithymia and a score lower than 51 indicates absence of alexithymia [3]. The TAS-20 has been translated and validated in Greek [15].

The HADS is a self-report rating scale of 14 items, designed to measure anxiety (HADS-A) and depression (HADS-D), with each subscale consisting of 7 items. It is worth noting that items referring to depressive symptoms that describe somatic aspects of depression (e.g. insomnia and weight loss) are not included in the scale. Thus, this scale is often used for the assessment of depression and anxiety in patients with physical illnesses. The HADS has been translated and validated in Greek [16]. In this study, a cutoff score above 8 is used to indicate the possible presence of anxiety or depression, which is suggested for detecting them in general population and in somatic patient samples [16].

The severity of psoriasis was assessed by a dermatologist using the Psoriasis Area and Severity Index (PASI score) [17]. The PASI incorporates the clinical extent of psoriasis (surface area of skin affected) and clinical severity of its manifestations (erythema, desquamation and induration). In this study, a score above 10 is used to diagnose severe psoriasis. The mild or moderate form of psoriasis is given a score of less than 10.

### Statistical analysis

Normality was assessed by Kolmogorov-Smirnov’s *Z* test. Descriptive statistics were measured and independent sample *t* tests were performed in order to examine whether there were any significant differences in the TAS total score and in its three subscales’ scores, as well as in the HADS total score and in its two subscales’ scores between patients with psoriasis and the control group and between male and female participants. Chi-square tests were applied for comparisons between male and female participants, HADS-A subscale and TAS scores and HADS-D subscale and TAS scores. Correlations were measured by Pearson’s *r* correlation coefficient between TAS, its subscales and age and between TAS and HADS and their subscales. A series of linear regression analyses were performed for the psoriatic patients’ group, in order estimate the prediction of alexithymia and its constitutes, DIF, DDF and EOT (dependent variables) from gender, age, severity of psoriasis, depression or anxiety. Statistical significance level was set at *p* < 0.05 (corrected where applicable) and statistical analyses were conducted using SPSS software package ver. 17.00 (SPSS Inc., Chicago, IL, USA).

## Results

The current study included 108 patients with psoriasis, 52 male and 56 female (*x*^2^ = 0.15, *df* = 1, *p* = 0.7 NS). The mean age of the patients was 52.53 (±16.41) years. Regarding alexithymia’s prevalence in the patients with psoriasis based on the aforementioned TAS-20 standards, 35 (32.4%) patients were classified as alexithymic, 24 (22.2%) as borderline and 49 (45.4%) as nonalexithymic. The mean TAS score was 52.62 (±13.46) for the patients and 39.63 (±10.20) for the controls.

Twenty-five of the total 108 patients were diagnosed with severe psoriasis (PASI score > 10) and presented a mean TAS score of 55.56 ± 13.23, which did not differ significantly from the total patients’ mean TAS score (*t* = −1.24, *df* = 106, two-tailed *p* = 0.22, NS). There was no significant correlation between the severity of psoriasis (PASI score) and alexithymia (*r* = 0.22, *p* > 0.05, NS).

The total group of patients with psoriasis compared with the 100 healthy controls (48 males and 52 females) matched for age (*t* = 1.92, *df* = 206, *p* > 0.05 NS) and gender (*x*^2^ = 0.014, *df* = 1, *p* = 0.90 NS) had significantly higher levels of alexithymia (*t* = 7.88, *p* < 0.001) (Table 1). As far as depression and anxiety are concerned, there were no significant differences between psoriatic patients and healthy controls (Table 1).

**Table 1:** **Descriptive statistics and comparisons between patients with psoriasis and healthy controls**

	**Patients**	**Control group**	***t*** **test**	***p*** **value**
TAS	52.62 ± 13.46	39.63 ± 10.21	7.88	0.001*
DIF	17.49 ± 6.27	13.69 ± 5.38	4.70	0.001*
DDF	13.72 ± 4.93	10.74 ± 4.69	4.46	0.001*
EOT	21.34 ± 5.51	15.59 ± 4.60	8.19	0.001*
HADS	10.99 ± 7.03	10.05 ± 5.52	1.07	0.28
HADS-D	5.16 ± 3.83	4.76 ± 2.93	0.86	0.39
HADS-A	5.82 ± 4.01	5.29 ± 3.34	1.04	0.30

Values are presented as mean ± SD.

Independent *t* tests in order to determine possible differences in TAS and HADS and their subscales between the mean scores of patients with psoriasis and control group.

*Statistically significant difference.

There were no any significant differences in the TAS total score and in its subscales’ scores, DIF, DDF and EOT, as well as in the HADS total score and in its subscales’ scores, HADS-A and HADS-D, in the psoriatic patients’ group between male and female participants as indicated by the independent sample *t* tests and thus these results are not presented. Concerning age, it was revealed that it was positively correlated with EOT, but not with TAS, DIF or DDF (Table 2).

**Table 2:** **Correlations between TAS, its subscales and age and TAS and HADS and their subscales (** ***n*** **= 108,** ***df*** **= 106)**

	**Age (** ***r*** **)**	**HADS (** ***r*** **)**	**HADS-A (** ***r*** **)**	**HADS-D (** ***r*** **)**
TAS	0.15	0.28**	0.29**	0.20*
DIF	0.03	0.29**	0.30***	0.20***
DDF	0.08	0.24**	0.30**	0.14^a^
EOT	0.29***	0.14^1^	0.10^a^	0.15^a^

Correlations by Pearson’s *r* correlation coefficient in the psoriatic patients’ group.

*p < 0.05; **p < 0.01; ***p < 0.001.

^a^Nonsignificant.

In the HADS-D subscale, 24% of the patients scored above the cutoff score and 65.4% of them also presented alexithymia (*x*^2^ = 63.63, *df* = 1, *p* = 0.000). Additionally, 31.5% of the patients scored above the cutoff score in the HADS-A and 61.8% of them were also alexithymic (*x*^2^ = 56.74, *df* = 1, *p* = 0.000).

The HADS and both its subscales were significantly positively correlated with the TAS total score, as well as with its subscale DIF. The HADS total score and HADS-A subscale were also significantly positively correlated with DDF, whereas EOT was not correlated with either HADS total score or its subscales (Table 2).

A series of linear regression analyses were performed for the psoriatic patients’ group in order to determine whether gender, age, severity of psoriasis, anxiety or depression could predict alexithymia or its main characteristics, DIF, DDF and EOT (Table 3). The linear regression analyses revealed that the age of the patients was a significant predictor only of EOT explaining 8% of its variation, whereas neither gender nor the severity of psoriasis (PASI) were able to predict any of the dependent variables. Anxiety contributed significantly to the prediction of alexithymia, DIF and DDF, explaining 9%, 10% and 9% of their variation, respectively. Depression contributed significantly to the prediction of alexithymia, explaining 4% of its variation, and of DIF and explaining 4% as well, but not of DDF. Finally, the prediction of EOT from anxiety and depression was not significant (Table 3).

**Table 3:** **Linear regression analyses for TAS, DIF, DDF and EOT in patients with psoriasis**

**Dependent variable**	**Predictors**	***R*** ^**2**^	***F***	***r***	***β***	***p*** **value**
TAS	Gender	0.005	(1, 106) = 0.52	1.86	0.07	0.48
	Age	0.02	(1, 106) = 2.57	0.13	0.15	0.11
	PASI	0.01	(1, 106) = 0.56	3.83	1.25	0.22
	HADS-A	0.09	(1, 106) = 10.02	0.99	0.30	0.002*
	HADS-D	0.04	(1, 106) = 4.27	0.69	0.20	0.04*
DIF	Gender	0.03	(1, 106) = 2.85	2.02	0.16	0.09
	Age	0.001	(1, 106) = 0.08	0.01	0.28	0.78
	PASI	0.003	(1, 106) = 0.29	0.77	0.05	0.60
	HADS-A	0.10	(1, 106) = 11.33	0.49	0.31	0.001*
	HADS-D	0.04	(1, 106) = 4.30	0.32	0.20	0.04*
DDF	Gender	0.001	(1, 106) = 0.14	−0.35	−0.04	0.71
	Age	0.007	(1, 106) = 0.73	0.03	0.08	0.40
	PASI	0.01	(1, 106) = 1.23	1.25	0.11	0.27
	HADS-A	0.09	(1, 106) = 10.08	0.36	0.30	0.002*
	HADS-D	0.02	(1, 106) = 2.03	0.18	0.14	0.16
EOT	Gender	0.001	(1, 106) = 0.002	−0.04	−0.004	0.97
	Age	0.08	(1, 106) = 9.68	0.10	0.30	0.002*
	PASI	0.01	(1, 106) = 1.20	1.38	0.11	0.28
	HADS-A	0.01	(1, 106) = 1.14	0.14	0.10	0.29
	HADS-D	0.02	(1, 106) = 2.57	0.22	0.15	0.11

Linear regression analyses for TAS and its subscales from demographic variables, PASI, HADS-A and HADS-D in patients with psoriasis.

*Statistically significant difference.

## Discussion

The purposes of the current study were (a) to examine the prevalence of alexithymia among patients with psoriasis, in comparison with healthy individuals from the general population and (b) to assess the association between depressive and anxiety symptoms and alexithymia, as well as its three dimensions, by taking into consideration demographic and clinical data of the patients.

The present study confirms that the prevalence of alexithymia is higher in patients with psoriasis than that in healthy individuals from the general population. This finding suggests that psoriasis could be ranked among the diseases associated with the deregulation of emotion, while the lack of recognition and expression of emotions might be an important aggravating factor in the appearance of the disease. This result is in accordance with most studies [11,13] although some researchers do not confirm this hypothesis [10].

In our study, there were no significant differences in the TAS score or in its subscales by gender, regarding patients with psoriasis. The majority of studies, mainly in the general population, reveal a higher prevalence of alexithymia, as a personality trait, in male than female individuals [6].

Regarding the possible effect of the severity of psoriasis on alexithymia, no significant differences of alexithymia and its dimensions between patients with severe and not severe psoriasis were observed, as classified by the PASI score. Moreover, there was no significant correlation between the severity of psoriasis and TAS score either. Richards and colleagues [18] did not find any correlations between alexithymia’s score and age, age onset, disease duration and clinical severity of psoriasis either [18].

As far as anxiety and depression are concerned, no significant differences were detected between patients with psoriasis and healthy controls. A similar finding was reported by Magin and colleagues, who have also used HADS to evaluate depression and anxiety [19]. While some previous studies have reported that patients with psoriasis were more likely to have higher depression levels than the general population, this may be due to the fact that they have used psychometric instruments containing somatic aspects, such as the Beck Depression Inventory, which may also be common in psoriasis [20]. Furthermore, as far as anxiety levels are concerned, conflicting findings have been reported when comparing psoriatic patients with healthy controls [19]. Nevertheless, despite the fact that depression and anxiety did not differ significantly between patients with psoriasis and healthy controls, it is noteworthy that 24% and 31.5% of the psoriatic patients scored above the cutoff score in the HADS-D and HADS-A subscale, respectively, a finding that is consistent with previous studies and which suggests that psychological morbidity is a clinically important concern in patients with psoriasis [19,21].

Additionally, a considerable rate of the psoriatic patients with depression was also alexithymic (65.4%). Several studies show the positive relationship between alexithymia and depression [22]. The same applies to anxiety, as the majority of the psoriatic patients with anxiety (61.8%) demonstrated increased levels of alexithymia. It seems that certain personality factors may make psoriatic patients more vulnerable to stress and thus contribute to the stress reactivity of the disease [10].

The aforementioned association between alexithymia, depression and anxiety is also confirmed by the correlations that were conducted between TAS and HADS, which revealed that both anxiety and depression were positively correlated with alexithymia. Furthermore, it was found that higher anxiety and depression were positively correlated with higher DIF, which might indicate that increased anxiety and depression in psoriatic patients may lead them to the difficulty in identifying feelings and distinguishing them from bodily sensations. In addition, the significant correlation that was found between HADS-A and DDF indicated that the psoriatic patients with anxiety symptoms, apart from their difficulty in identifying and understanding their feelings were not able to communicate them either. This might indicate that alexithymia plays a role in emotional avoidance [23]. Several studies in different patient populations reveal that depression and anxiety are related to the TAS subscales either DIF or/and DDF and via them to the total score of TAS [5,23,24].

Similar findings are suggested by the linear regression analyses. The analyses indicated that psoriasis constitutes a significant predictor for the performance in the TAS, regardless of the gender, age and the severity of psoriasis. Anxiety mainly and, to a lesser degree, depression are significant predictors of alexithymia. Both anxiety and depression contribute significantly to the prediction of DIF, while anxiety to the prediction of DDF as well. On the contrary, EOT was significantly predicted only from the age of the patients. Bonnet and colleagues [5] found significant relationships between affective symptoms and the emotional dimensions (DIF and DDF) of alexithymia. In the same study, however, they found that only anxiety contributed significantly to the aforementioned two dimensions of alexithymia [5,23]. Leweke and colleagues using multiple hierarchical regression suggested that higher TAS scores were associated with anxiety and depression disorders and that DDF subscale was significantly related to depressive disorder [25].

It has been found that EOT is not related to depression and anxiety [23]. This is confirmed in our study as well, since only age contributed significantly to the prediction of EOT. This, along with the positive correlation between age and EOT, implies that as the years pass, the psoriatics in our sample become rather dependent on external reality and unable to attribute the causes of their symptoms to emotional. Tolmunen and colleagues report that a higher age is independently associated with increase in TAS scores [26].

Our results indicate that alexithymia in patients with psoriasis is independent from the age, the gender and the severity of the disease, but there is a significant association between anxiety or/and depression and alexithymia. This relationship is mainly due to its subscales rather than to the total alexithymia per se. According to these results, alexithymia does not seem to be a stable condition among our psoriatic patients with anxiety and depressive symptoms. Nevertheless, alexithymia seems to be independent of the age, the gender and the severity of illness among the psoriatic patients. Therefore, alexithymic individuals, who have difficulties in communicating their feelings, are at risk of developing specific diseases. Therefore, Allegranti and colleagues recommended psychotherapy for dermatologic alexithymic patients to help them recognize their feelings and use them as signals of emotional stress [7].

Some limitations of our study should be mentioned. In spite of the fact that the present study in comparison with previous ones uses a rather high sample, this could be larger in order to ensure that its findings could be safely generalized. Another limitation is that there was no follow up or a repeated examination.

## Conclusions

In conclusion, the current study revealed that the prevalence of alexithymia in patients with psoriasis is higher than that in healthy individuals from the general population. Nevertheless, they do not present significant differences in depression or anxiety levels. Furthermore, there is no significant difference in alexythimia’s levels between male and female psoriatic patients. Higher anxiety and depression levels are associated with higher alexithymia, which is independent of gender, age and severity of psoriasis. Regarding alexithymia’s dimensions, difficulty in identifying feelings is associated with both anxiety and depression, whereas difficulty in describing them is related only with anxiety. On the contrary, externally oriented thinking is independent of both anxiety and depression, but it is predicted only from age. To our knowledge, no other case–control study exists addressing the relationship between psoriasis and alexithymia that either uses such a sample’s size or takes into consideration depression and anxiety without including somatic aspects in their assessment.

Alexithymia should be taken into consideration by clinicians, in order to design appropriate interventions. The relationship between alexithymia and psoriasis in patients with psoriasis needs further investigation.

## Abbreviations

DIF: Difficulty in indentifying feelings
DDF: Difficulty in describing feelings
EOT: Externally oriented thinking
HADS: Hospital Anxiety and Depression Scale
TAS-20: Toronto Alexithymia Scale
HADS-D: Hospital Anxiety and Depression Scale—depression subscale
HADS-A: Hospital Anxiety and Depression Scale—anxiety subscale
PASI: Psoriasis Area and Severity Index
